# Case report: Severe deep ulcer on the left abdomen mimicking mycosis fungoides caused by *Trichophyton tonsurans* in a patient with novel CARD9 mutation

**DOI:** 10.3389/fimmu.2022.1015000

**Published:** 2022-09-28

**Authors:** Jingwen Tan, Qian Yu, Zhiqin Gao, Hong Yang, Qilong Chen, Lianjuan Yang

**Affiliations:** ^1^ Department of Medical Mycology, Shanghai Skin Disease Hospital, Tongji University School of Medicine, Shanghai, China; ^2^ Central Laboratory, Shanghai Skin Disease Hospital, Tongji University School of Medicine, Shanghai, China

**Keywords:** deep dermatophytosis, tinea corporis, *Trichophyton tonsurans*, novel CARD9 mutation, posaconazole

## Abstract

Dermatophytosis is the most common type of superficial fungal infection caused by dermatophytes. Occasionally, the fungus invades deep into the dermis or other tissues, causing deep dermatophytosis. Deep dermatophytosis is often associated with Caspase Recruitment Domain-containing protein 9 (CARD9) deficiency in patients. Here, we report the first case of deep dermatophytosis with a rare mycosis fungoides manifestation caused by *T. tonsurans* in a patient with a novel mutation in exon 4 of *CARD9*. The condition presented with heterozygous K196E mutation, which leads to deficiency of innate and adaptive immune responses in the patient, and caused intractable severe lesions. The patient received treatment with multiple antifungal drugs and was ultimately alleviated by posaconazole. These findings extend the pathogen spectrum of deep dermatophytosis linked with CARD9 deficiency and enriched their phenotypic spectrum.

## Introduction

Dermatophytosis is the most common type of superficial fungal infections caused by dermatophytes. Occasionally, the fungus invades deep into the dermis or other tissues, causing deep dermatophytosis (1). The most common pathogenic pathogen is *Trichophyton rubrum*, followed by *T. mentagrophytes*, *Microsporum canis*, *T. tonsurans*, *T. interdigital* and *T. violaceum* (1).

Deep dermatophytosis is a rare, invasive, sometime life-threatening fungal infection. This condition mainly occurs in immunosuppressed individuals or in patients with mutations in genes including Caspase Recruitment Domain-containing protein 9 (*CARD9*) and signal transducer and activator of transcription 3 (*STAT3*) (1–3). CARD9 is an essential adapter that plays an important role in antifungal immunity by regulating downstream pattern recognition receptor signaling. CARD9 deficiency can cause impaired immune response to fungal infections and lead to refractory severe infection.

Here, we reported a case of deep dermatophytosis with a rare mycosis fungoides manifestation caused by *T. tonsurans* in a patient with novel CARD9 mutation. The study was approved by the ethics committee of Shanghai Skin Disease Hospital.

## Case description

A 38-year-old male mental worker visited our clinic in November 2017 presenting a deep ulcer on his left abdomen. Ten years ago, several diffuse asymptomatic brown patches had developed on his trunk. These patches had been diagnosed as tinea versicolor based on KOH mount, but they showed no noticeable improvement with itraconazole and bifonazole treatment. One year ago, a brown patch at his left abdomen had been chafed by the safety belt during a car accident, causing the development of a dark red plaque with ulcer on his left belly with moderate pain. A similar lesion appeared on his left breast several months later. The rest of his medical and family history was unremarkable.

**Figure 1:**
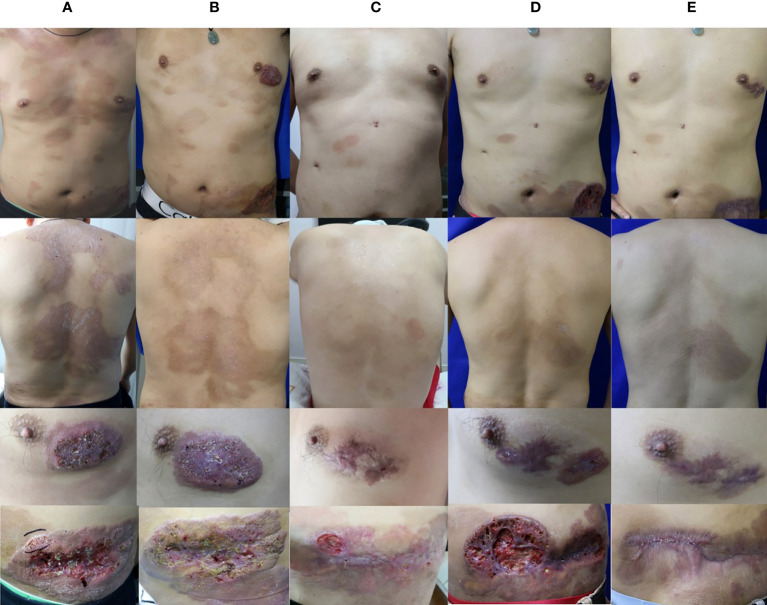
Clinical image. **(A)** Skin lesions of the patient during the initial visit. **(B)** Skin lesions of the patient after 4 months of oral terbinafine and itraconazole treatment. **(C)** Skin lesions of the patient after 15 months of combination treatment with oral terbinafine, itraconazole, and ALA-PDT. **(D)** Skin lesions of the patient after 14 months of oral voriconazole. **(E)** Skin lesions of the patient after 4 months of oral posaconazole.

Physical examination revealed generalized, discrete, brown patches with furfural scales on his trunk. His left breast showed an irregular dark red plaque of size 3×5 cm covered by scab. Dark red plaque of about 10×20 cm size with a 3×10 cm linear deep ulcer in the center was also present on his left abdomen. The ulcer had an irregular edge, and its lower portion was covered with scab and contained pus (**Figure 1A**). Laboratory examination and radiological investigation ruled out underlying malignancies and human immunodeficiency virus infection. The case was initially considered as that of mycosis fungoides. Histopathology examination was performed for confirmation.

**Figure 2:**
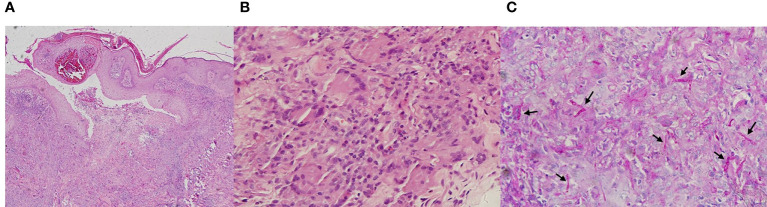
Pathological diagnosis of deep dermatophytes. **(A, B)** Hematoxylin and eosin staining of the deep dermatophytes revealed pseudoepitheliomatous hyperplasia; neutrophilic microabscesses, epithelioid cells, and multinucleated giant cells were observed within the dermis (Magnification: **A**, 40×; **B**, 400×). **(C)** Periodic acid–Schiff stains showed septate fungal hyphae (black arrow) in the stratum corneum or within giant cells (Magnification: 400×).

**Figure 3:**
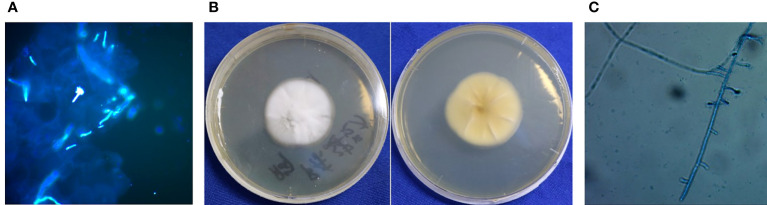
Results of etiological examination. **(A)** Direct fluorescence of multiple skin lesions were positive for pseudohyphae (Magnification: 40×). **(B)** Macroscopic appearance of *T. tonsurans*: white, villiform colony on SDA after 7 d of incubation at 28°C. **(C)** Microscopic examination of the morphology of mycelium.

The examination revealed pseudoepitheliomatous hyperplasia. Neutrophilic microabscesses, epithelioid cells, and multinucleated giant cells were observed within the dermis (**Figures 2A, B**). Periodic acid–Schiff stains showed septate fungal hyphae in the stratum corneum or within giant cells (**Figure 2C**). Direct microscopic examination of both the brown patch and the ulcer were positive for the fungal hyphae (**Figure 3A**), and tissue culture isolated a white, villiform colony on Sabouraud Dextrose Agar after seven days of incubation at 28°C (**Figure 3B**). Microscopic examination of the culture revealed hyphae with membranes and lateral interval growth of rod-shaped conidium (**Figure 3C**). Internal transcribed spacer region nucleotide sequencing further confirmed the strain as *T. tonsurans* (GenBank Accession No. MW301352). The patient was diagnosed with deep dermatophytosis and tinea corporis caused by *T. tonsurans*.

*In vitro* susceptibility was tested following the Clinical and Laboratory Standards Institute (CLSI) M38-A2 protocol (4). The minimum inhibitory concentration (MIC) was determined by 80% inhibition compared with the growth control. The MIC values was 0.125μg/ml for itraconazole (ITC), 0.06 μg/ml for voriconazole (VRC), 0.25μg/ml for posaconazole (POS) and 0.03 μg/ml for terbinafine (TRB).These results indicated that this isolate was sensitive to common antifungal agents. The patient was then started on oral terbinafine therapy (250 mg daily). The lesion showed slight improvement after three months of treatment, and oral itraconazole (400 mg daily) was started simultaneously. After one month of combined therapy, the treatment for the lesion showed a good progress (**Figure 1B**). Considering the side effects of high-dose antifungal drugs, the therapeutic regimen was changed to only oral itraconazole (400 mg daily), and continued for seven months. During this period, the patient took short-term antibacterial agents, ALA–photodynamic therapy (ALA-PDT), and water-filtered infrared-A (wIRA) as adjuvants. Although the brown patches mostly disappeared and the ulcers showed partial healing, the plaques on both breast and abdomen persisted, and *T. tonsurans* could still be isolated. The patient was then administered combined oral itraconazole (400 mg daily) and terbinafine (250 mg daily) therapy for eight months (**Figure 1C**); these lesions remained unresponsive. Then, the therapeutic regimen was changed to voriconazole (400 mg daily), according to the *in vitro* antifungal susceptibility test results. Initially, the dark red plaques flattened considerably and majority of the brown patches disappeared. However, after several months, the lesion showed no further change, and *T. tonsurans* could still be isolated. Then, we tried topical amphotericin B with hydropathic compress and the ulcer on the abdomen relapsed. The skin lesions continued to deteriorate, nearly penetrating through the abdominal wall (**Figure 1D**). We changed the antifungal agent to posaconazole (400 mg daily). The condition of the lesion improved gradually and the ulcers healed after four months of this therapeutic regimen (**Figure 1E**). The integral therapeutic process and clinical outcome images are shown in supplementary material (**Supplementary Figure**).

**Figure 4:**
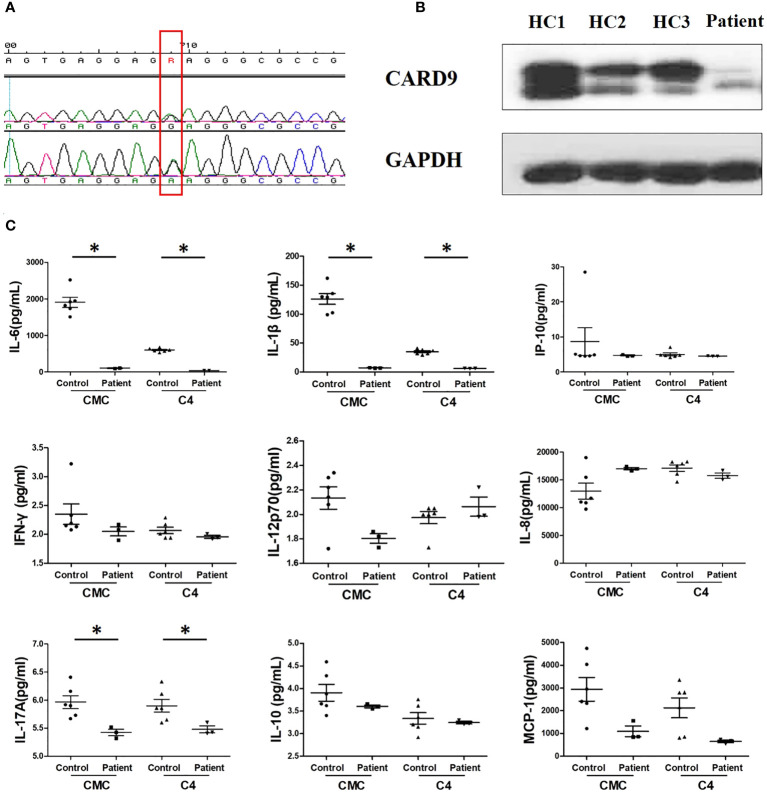
Results of immunological examination. **(A)** Sanger sequencing of *CARD9* exon 4 reveals the heterozygous replacement of c.596A>R (red frame); F, forward chain; R, reverse chain. **(B)** Western blot of PBMCs showed decreased CARD9 levels, compared to healthy control; HC1, HC2, HC3, healthy control **(C)** Comparison of IL-1β, IL-6, and IL-17A production between the patient and healthy control after stimulation with heat-killed isolates of patient strain (C4) and *T. tonsurans* wild-type strain (CMC), *P < 0.05, differences between groups were analyzed using Mann–Whitney U test. *P-*values reported in the study were based on a two-sided test.

We sequenced the *CARD9* gene of the patient. Exon 4 showed a novel c.596A>R (K196E) mutation (**Figure 4A**). Western blot of peripheral blood mononuclear cells (PBMCs) isolated from the patient showed decreased CARD9 levels, compared to that in healthy controls (**Figure 4B**). To confirm the functional effect of this novel mutation, the PBMCs of the patient were tested for the release of inflammatory cytokines in response to stimulations. IL-6, IL-1β and IL-17A levels were distinctly lower after stimulation, compared that in healthy controls (**Figure 4C**).

## Discussion

Deep dermatophytes typically show an indolent clinical course and unique pathological manifestations of fungal infection in association with superficial dermatophytosis. Clinical presentation varies and may depend on many factors, including the site of infection, host immunity, and virulence of dermatophytes. In this case, the initial lesions presented with non-inflammatory brown patches without any other symptom, eventually broke through skin barrier and developed into severe, huge, and deep ulcer. The patient did not respond to common antifungal agents, though culture sensitivity tests revealed their *in vitro* susceptibility to antifungals. Therefore, we speculated that he had antifungal immunodeficiency. The *CARD9* mutation was detected by sequencing the PBMCs, which is associated with fungal infections.

CARD9 is a critical adapter in antifungal immunology. It is mainly expressed in myeloid cells and principally involved in pattern recognition by C-type lectin receptors (CLRs) (5). People with CARD9 deficiency are mainly healthy; however, they can be more susceptible to fungal infections. There are only 21 reported cases of deep dermatophytes infection in eight countries associated with CARD9 deficiency so far (2, 3, 6–12), wherein *T. rubrum* accounts for more than 50% of infections. The homozygous mutation p.Q289X was detected in most cases (2). To our knowledge, this is the first report of deep dermatophytosis caused by *T. tonsurans* linked with CARD9 deficiency. Based on *in vitro* experiments, we confirmed that the CARD9 mutation can inhibit the release of inflammatory cytokines, such as IL-6, IL-1β, and IL-17A, which possibly makes it difficult for monocytes to trigger inflammatory reactions *in vivo*.

However, though susceptible to both drugs, posaconazole treatment showed better results than that with voriconazole. Previous reports indicate the presence of antifungal drugs to be a possible contributing factor for their side effects of impaired polymorphonuclear neutrophils (PMN) functionality in immune cells. Voriconazole can decrease the formation of neutrophil extracellular traps (NETs) and antifungal drug-induced impaired PMN mortality. Posaconazole can activate PMN effect and enhance the generation of reactive oxygen species, formation of NETs, and degranulation; therefore, it can improve this aspect of antifungal therapy (13). Thus, we chose posaconazole to successfully treat the current patient. When the patients with deep dermatophytosis show no response to common antifungal agents, the immunosuppressive effect of these drugs themselves should be considered.

In conclusion, we reported the first case of extensive deep dermatophytosis with rare clinical manifestations, like mycosis fungoides, caused unusually by *T. tonsurans*. These findings added to the pathogenic spectrum of deep dermatophytosis. This patient possessed novel CARD9 K196E mutation, which enriched the phenotypic spectrum of CARD9 deficiencies. When dermatophytosis presents with extensive and chronic non-inflammatory or severe refractory lesions, the possibility of CARD9 deficiency in the patient should be investigated.

## Data availability statement

The original contributions presented in the study are included in the article/**Supplementary Material**. Further inquiries can be directed to the corresponding author.

## Ethics statement 

The studies involving human participants were reviewed and approved by the ethics committee of Shanghai Dermatology Hospital. The patients provided written informed consent for participation in the study and publication of potentially identifiable images or data included in this article.

## Author contributions

JT and QY: design and drafting of the work, analysis, acquisition, and interpretation of data. ZG and HY: laboratory testing and data analysis. QC: design of experimental and interpretation of data. LY: study design, revision, and finalization of the manuscript. All authors contributed to the article and approved the submitted version.

## Funding

This work was supported by National Natural Science Foundation of China (Grant No. 82102418 to JT, Grant No. 82173429 to LY), Shanghai Municipal Commission of Health and Family Planning (Grant No. 201940476 to LY, Grant No. 20194Y0337 to QY) and Technology Commission of Shanghai Municipality (Grant No. 18411969700 and 20Y11905600 to QY, Grant No. 21Y11904900 to LY).The funders had no role in study design, data analysis, decision to publish, or preparation of the manuscript.

## Acknowledgments

We thank the patient, the family members, and healthy donors for their participation in this study. We thank Professor Hai Wen from Department of Dermatology, Shanghai Changzheng Hospital, Second Military Medical University, Shanghai, China, Professor Ruoyu Li and Xiaowen Wang from Peking University First Hospital, Research Center for Medical Mycology, Peking University, Beijing, Professor Xiaowen Huang from Department of Dermatology, Nanfang Hospital, Southern Medical University, Guangzhou, China for pathologic diagnosis, patient therapy assistance, and comments on the manuscript.

## Conflict of interest

The authors declare that the research was conducted in the absence of any commercial or financial relationships that could be construed as a potential conflict of interest.

## Publisher’s note

All claims expressed in this article are solely those of the authors and do not necessarily represent those of their affiliated organizations, or those of the publisher, the editors and the reviewers. Any product that may be evaluated in this article, or claim that may be made by its manufacturer, is not guaranteed or endorsed by the publisher.

## Supplementary material

Supplementary Figure The integral therapeutic process of the patient.
