# Supplementary figures and images for: Case report: Severe deep ulcer on the left abdomen mimicking mycosis fungoides caused by Trichophyton tonsurans in a patient with novel CARD9 mutation
Source: Front Immunol. 2022 Sep 28;13:1015000. doi: 10.3389/fimmu.2022.1015000 (PMC9554596; doi:10.3389/fimmu.2022.1015000)

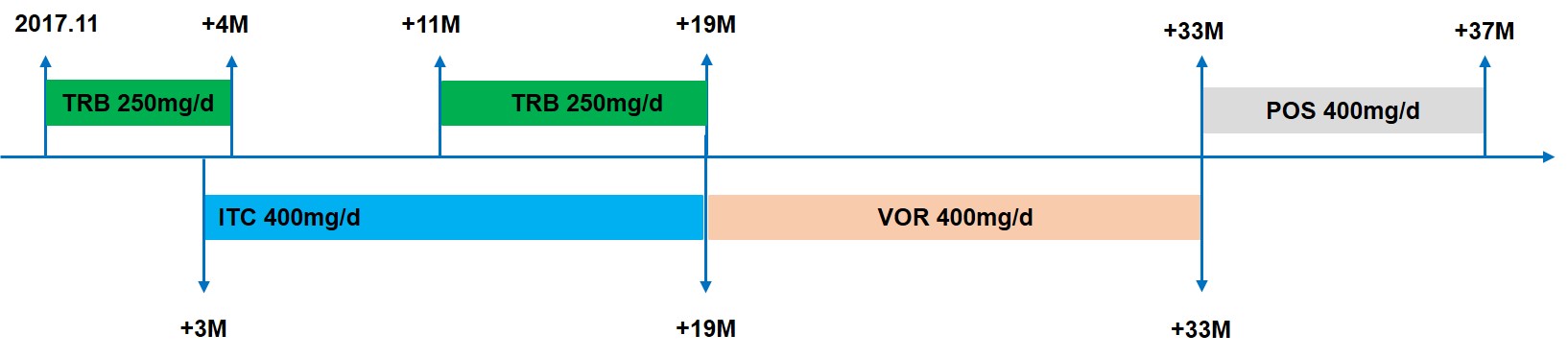

Supplement: Supplementary Figure — The integral therapeutic process of the patient. [file Image_1.jpeg]
